# MRI-based 2.5D deep learning radiomics nomogram for the differentiation of benign versus malignant vertebral compression fractures

**DOI:** 10.3389/fonc.2025.1603672

**Published:** 2025-05-14

**Authors:** Wenhua Liang, Hong Yu, Lisha Duan, Xiaona Li, Ming Wang, Bing Wang, Jianling Cui

**Affiliations:** Department of Radiology, Third Hospital of Hebei Medical University, Shijiazhuang, China

**Keywords:** radiomics, 2.5D deep learning, feature fusion, vertebral compression fractures, nomogram, magnetic resonance imaging

## Abstract

**Objective:**

Vertebral compression fractures (VCFs) represent a prevalent clinical problem, yet distinguishing acute benign variants from malignant pathological fractures constitutes a persistent diagnostic dilemma. To develop and validate a MRI-based nomogram combining clinical and deep learning radiomics (DLR) signatures for the differentiation of benign versus malignant vertebral compression fractures (VCFs).

**Methods:**

A retrospective cohort study was conducted involving 234 VCF patients, randomly allocated to training and testing sets at a 7:3 ratio. Radiomics (Rad) features were extracted using traditional Rad techniques, while 2.5-dimensional (2.5D) deep learning (DL) features were obtained using the ResNet50 model. These features were combined through feature fusion to construct deep learning radiomics (DLR) models. Through a feature fusion strategy, this study integrated eight machine learning architectures to construct a predictive framework, ultimately establishing a visualized risk assessment scale based on multimodal data (including clinical indicators and Rad features).The performance of the various models was evaluated using the receiver operating characteristic (ROC) curve.

**Results:**

The standalone Rad model using ExtraTrees achieved AUC=0.801 (95%CI:0.693-0.909) in testing, while the DL model an AUC value of 0.805 (95% CI: 0.690-0.921) in the testing cohort. Compared with the Rad model and DL model, the performance superiority of the DLR model was demonstrated. Among all these models, the DLR model that employed ExtraTrees algorithm performed the best, with area under the curve (AUC) values of 0.971 (95% CI: 0.948-0.995) in the training dataset and 0.828 (95% CI: 0.727-0.929) in the testing dataset. The performance of this model was further improved when combined with clinical and MRI features to form the DLR nomogram (DLRN), achieving AUC values of 0.981 (95% CI: 0.964-0.998) in the training dataset and 0.871 (95% CI: 0.786-0.957) in the testing dataset.

**Conclusion:**

Our study integrates handcrafted radiomics, 2.5D deep learning features, and clinical data into a nomogram (DLRN). This approach not only enhances diagnostic accuracy but also provides superior clinical utility. The novel 2.5D DL framework and comprehensive feature fusion strategy represent significant advancements in the field, offering a robust tool for radiologists to differentiate benign from malignant VCFs.

## 1 Introduction

Vertebral compression fractures (VCFs) are a prevalent clinical condition characterized by acute or chronic back pain, functional disability, diminished quality of life, and elevated mortality rates (1, 2). While osteoporosis, mechanical trauma, and neoplastic infiltration constitute primary etiological factors (3), non-traumatic VCFs in elderly populations frequently manifest as osteoporotic fractures or tumor-related lesions (4, 5). Notably, distinguishing between benign and malignant VCFs remains diagnostically challenging due to overlapping imaging characteristics, particularly for clinicians with limited expertise (6). However, accurate diagnosis plays an important role in appropriate patient management.

Magnetic resonance imaging(MRI) is essential for evaluating spinal diseases. The superior soft tissue resolution of MRI has led to good performance at detecting bone marrow abnormalities, and its notable diagnostic efficacy for malignant vertebral compression fractures (VCFs) has been established in previous research (7). Various MRI features, such as signal intensity and mass, assist in differentiating acute benign VCFs from malignant ones. The T2-weighted Dixon image, which include image similar to fat-suppressed T2-weighted image, is a crucial element of MRI, especially for visualizing and characterizing lesions (8). However, these features are not definitive characteristics due to the similar variations and their complexity in marrow. As a result, differentiating benign from malignant VCFs can be challenging, especially in patients who do not exhibit typical MRI morphological features (9).

Radiomics (Rad) enables high-throughput extraction of quantitative imaging features for enhanced disease characterization. In orthopedic-related diseases, Rad has emerged as a powerful tool for decoding pathological patterns invisible to human eyes (10–13). Rad has shown promise in capturing tissue heterogeneity and providing objective biomarkers for diagnosis. Combined with machine learning algorithms, Rad features can be transformed into predictive models that enhance diagnostic accuracy beyond visual assessment alone. These successes highlight radiomics’ potential to augment traditional image interpretation in complex orthopedic diagnostics. Critical to model development, optimal sequence selection minimizes redundant feature extraction, mitigates overfitting risks, and ensures adequate sample sizes. These considerations motivated our selection of T2-weighted Dixon sequences for analysis (14).

Recently, deep learning (DL), particularly convolutional neural networks (CNNs), has revolutionized image recognition tasks by automatically learning hierarchical features from raw data. The application of DL to spinal imaging has demonstrated its potential in detecting and classifying VCFs (15–17). However, current technical limitations warrant attention. Predominant 2D DL methods neglect inter-slice contextual information, while 3D approaches demand prohibitive computational resources and large-scale datasets (18).

In this study, we developed a 2.5-dimensional (2.5D) DL by utilizing the maximum and adjacent sagittal slices of the VCFs, which represents a novel approach to analyze the VCFs. To our knowledge, no existing research has focused on utilizing fusion models of traditional Rad and 2.5D DL features for differentiating the type of VCFs based on MRI. We hypothesized that the fusion of 2.5D spatial information with handcrafted radiomics would outperform conventional radiomics or deep learning approaches alone. Our study aimed to develop a multimodal MRI-based nomogram integrating 2.5D deep learning radiomics (DLR) and clinical features to distinguish benign from malignant VCFs.

## 2 Materials and methods

### 2.1 Patients

**Figure 1:**
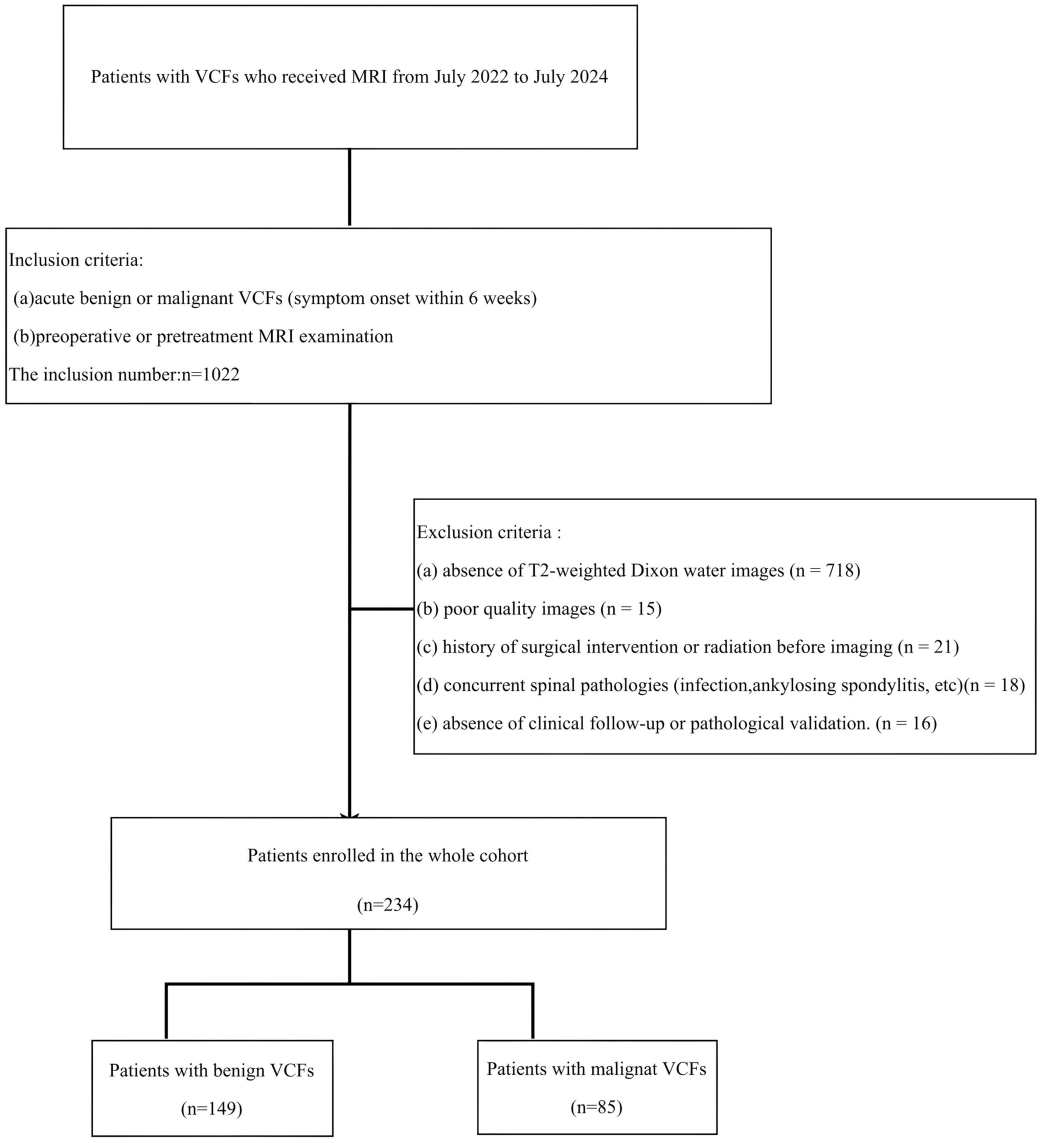
The inclusion and exclusion criteria for patients with VCF for the training and testing cohorts.

This retrospective study received institutional Ethics Review Board approval, with waived informed consent requirements. MRI examinations performed between July 2022 and July 2024 were systematically reviewed. Inclusion criteria required:(a) acute benign or malignant VCFs (symptom onset within 6 weeks); (b) preoperative or pretreatment MRI examination. Cases were excluded based on: (a) absence of T2-weighted Dixon water images; (b) poor quality images;(c) history of surgical intervention or radiation before imaging; (d) concurrent spinal pathologies (infection, ankylosing spondylitis, etc); (e) absence of clinical follow-up or pathological validation. The definitive diagnoses of vertebral compression fractures (VCFs) in all enrolled subjects were determined through comprehensive clinical evaluation. For benign fracture characterization, inclusion criteria required absence of prior oncologic history combined with follow and confirmation with stable disease. Conversely, malignant VCFs were confirmed through either biopsy or the known progression of related primary tumor diseases. The specific recruitment procedures are outlined in **Figure 1**.

**Figure 2:**
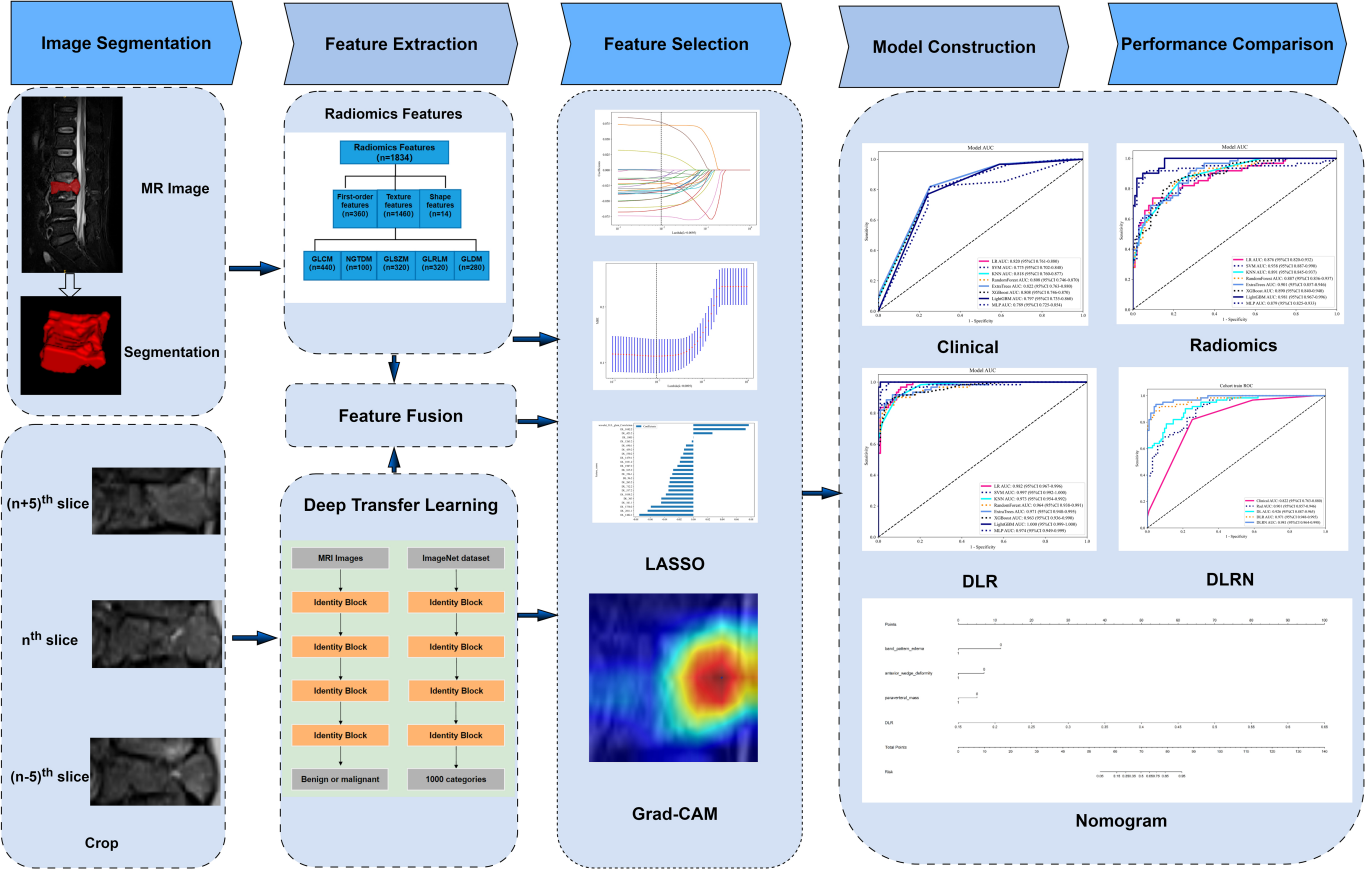
The overall flow chart of this study.

The dataset was stratified into training (70%) and testing (30%) subsets using random sampling. The technical workflow encompassing data preprocessing, model development, and validation phases are delineated in **Figure 2**.

### 2.2 Image acquisition and analysis

#### 2.2.1 MRI protocols

All examinations were conducted using two 3.0-T MRI platforms: Ingenia CX (Philips Healthcare, Amsterdam) or Signa Architect (GE Healthcare, Milwaukee). Four image series, including water, fat, in-phase, out-of-phase images were produced by the sagittal T2-weighted Dixon sequence, of them water Dixon (w-Dixon) images were selected for analysis due to their superior lesion contrast resembling fat-suppressed T2-weighted imaging. Acquisition parameters were as follows: TR, 2129–3862 ms; TE, 75–107 ms; field of view, 23–36 cm; matrix size, 180-192×256-320; slice thickness, 3–4 mm.

#### 2.2.2 Clinical data and MRI evaluation

In this study, all clinical information and MRI images of patients were retrieved from the Picture Archiving and Communication System (PACS). Clinical data included gender, age and location. The presence or absence of some MRI features were recorded, including anterior wedge deformity, band pattern edema, paravertebral mass, diffuse signal change, pedicle and posterior element involvement. Two board-certified musculoskeletal radiologists (D.L.S., 9 years’ experience; L.W.H., 11 years’ experience) independently interpreted all MRI examinations following standardized blinding protocols. This rigorous methodology ensured complete separation from clinical histories and histopathological results throughout the image analysis process. Following inter-reader reliability assessment between the two aforementioned radiologists, and features with agreement scores >0.75 were included in the subsequent analysis.

Gender, age and location of lesion were recorded in each case. If there were anterior wedge deformity, band pattern edema, paravertebral mass, diffuse signal change, pedicle and posterior element involvement of involved vertebral body were interpretated separately by two musculoskeletal radiologists with substantial clinical experience (D.L.S.: 9 years; L.W.H.: 11 years), with strict adherence to blinding protocols that excluded access to clinical histories and histopathological findings.

### 2.3 Image preprocessing and segmentation

N4-bias field correction was applied to all MRI data, followed by resampling to a uniform voxel size of 1×1×1 mm³ using nearest-neighbor interpolation. Subsequently, image signal intensities were normalized. Three-dimensional segmentation was performed using ITK-SNAP version 3.6.0 (www.itksnap.org/) on w-Dixon images to delineate the region of interest (ROI).The entire compressed vertebral bodies are the ROIs in our study. Radiologist D.L.S. conducted the initial segmentation. A randomly selected subset of 100 patient records was analyzed to assess intra-observer and inter-observer agreement reliability. Two weeks later, both Radiologist D.L.S. and Radiologist L.W.H. independently performed ROI delineation on this subset of data.

### 2.4 Rad feature extraction

The extraction of radiomic features was performed utilizing a in-house feature analysis tool, which was built upon the Pyradiomics platform (accessible at http://pyradiomics.readthedocs.io). The manually engineered radiomic features comprised three fundamental categories: (1) statistical features, (2) morphological characteristics, and (3) textural patterns. Various features a reclassified into texture group, such as greyscale co-occurrence matrix (GLCM), grey-level size zone matrix (GLSZM), grey-level run length matrix (GLRLM), and neighboring grey-tone difference matrix (NGTDM) and grey level dependence matrix (GLDM). In total, 1835 handcrafted features were extracted.

### 2.5 2.5DDL feature extraction

The 2.5D approach was chosen to balance computational efficiency and 3D spatial context. Unlike 2D models that discard inter-slice relationships, our method combined the maximum sagittal slices along with their adjacent slices (± 5 slices) into three separate input channels while avoiding the prohibitive GPU memory requirements of full 3D CNNs. Our 2.5D method utilized a total of 702 (234 × 3) images. Prior to model training, all input images underwent preprocessing steps involving two key operations: (1) spatial cropping to retain the minimal bounding box containing the target region, and (2) intensity normalization through Z-score transformation to standardize pixel values. This process simplified the images, reducing the complexity and background noise for the algorithm to analyze. During the model optimization phase, the synthetically generated 2.5D datasets were systematically integrated into a transfer learning paradigm, allowing for comprehensive evaluation of their augmentative effects on cross-domain adaptation capabilities. Transfer learning allows a model to enhance learning efficiency and minimizes the likelihood of overfitting. We explored the performance of ResNet50 architectures that had been pre-trained on the ImageNet Large Scale Visual Recognition Challenge 2012 (ILSVRC-2012) dataset. After examining the 702 images, we configured the DL parameters as follows: 50 training echoes and a batch size of 32. Grayscale intensities were normalized to the [-1,1] range via min-max scaling during preprocessing. All cropped regions were then resampled to 224×224 resolution with nearest-neighbor interpolation prior to model input.

### 2.6 Feature fusion and model construction

Rad feature analysis was performed through a multi-stage pipeline to ensure methodological rigor. In the preliminary phase, the reliability of features was evaluated through intraclass correlation coefficient (ICC) analysis. Features exhibiting ICC scores below the predefined threshold of 0.85 were systematically excluded to ensure robust measurement consistency across repeated evaluations. Subsequently, Z-score normalized features underwent statistical screening through inter-group t-tests, where features exhibiting statistically significant differences (p<0.05) were selected. To address feature redundancy, pairwise Pearson correlations were computed, and highly correlated feature pairs (r>0.9) were reduced to single representative features. A stepwise recursive elimination algorithm was then applied to iteratively remove collinear features. Finally, least absolute shrinkage and selection operator (LASSO) regression with 10-fold cross-validation was implemented to optimize regularization parameters (λ), ultimately retaining features with non-zero coefficients as the final robust predictors demonstrating superior discriminative performance. We extracted the DL features from the ResNet50 module. We then extracted a total of 2048-dimensional deep conventional neural network (DCNN) features for each of the three channels from the last average pooling layers. For the feature fusion, we composed Rad features and DL features including 2048-dimensional deep features for each of the three channels.

To construct radiomics and deep learning radiomics (DLR) models for distinguishing between benign and malignant VCFs, a feature selection process was implemented to identify relevant Rad features and their fused counterparts. These attributes were later combined with eight distinct machine learning classifiers, encompassing logistic regression (LR), support vector machines (SVM), k-nearest neighbors (KNN), Random Forest, ExtraTrees, XGBoost, light gradient boosting machine(LightGBM), and multilayer perceptron (MLP) architectures. To enhance the model’s hyperparameter tuning, a 5-fold cross-validation approach was systematically applied to the training dataset, enabling the identification and selection of classifiers demonstrating superior performance metrics for final model training.

After an exhaustive evaluation of pertinent clinical variables and MRI characteristics, an initial univariate screening process was performed to isolate potential predictive factors. Following this phase, multivariate logistic regression analysis was implemented to enhance feature selection precision. The optimized feature subset was systematically fused with the top-performing prediction model, ultimately producing an interpretable clinical nomogram to guide diagnostic evaluations.

The diagnostic accuracy of predictive models was evaluated through receiver operating characteristic (ROC) analysis, where the Delong statistical method was applied to determine significant differences in area under the curve (AUC) values across comparative models. Predictive calibration was quantitatively assessed using probability calibration diagrams supported by Hosmer-Lemeshow goodness-of-fit testing, which statistically evaluates the consistency between model-generated probabilities and actual outcome distributions. Simultaneously, decision curve analysis (DCA) was implemented to appraise clinical applicability and net benefit thresholds of the predictive models.

### 2.7 Statistical analysis

This study implemented Python (version 3.7.12) and the stats models library (version 0.13.2) for statistical computations, while machine learning models were developed using the scikit-learn API (version 1.0.2).Training of deep learning models was executed on an NVIDIA 4080 GPU, leveraging the MONAI framework (v. 0.8.1) and PyTorch (v. 1.8.1).

Statistical analyses were conducted to evaluate the normality distribution and variance homogeneity of quantitative datasets. For measurements adhering to normal distribution patterns, descriptive statistics were presented as means with standard deviations (SD), while between-group differences were assessed through independent samples t-tests for dual-group comparisons. Conversely, if the data deviated from normality, they were represented by the median and interquartile range(IQR), and comparisons were conducted using the non-parametric Mann-Whitney U test. For categorical data, comparisons were made using the chi-square test. Statistical significance was determined at a P-value threshold of <0.05.

## 3 Results

### 3.1 Clinical and MRI features

Clinical data and MRI characteristics were compared among patients in both the training and testing cohorts. The inter-reader agreement for all MRI features was greater than 0.75. The clinical information and MRI features of the patients involved in training and testing the models are summarized in **Table 1**. There are no statistically significant differences between most of the clinical and MRI features of the two groups. In this investigation, a thorough univariate examination was performed on all clinical attributes, with particular emphasis on determining the odds ratios (OR) and their associated p-values for every variable. As shown in **Table 2**, the baseline characteristics of the training and testing cohorts were well-balanced, with no statistically significant differences (P>0.05) in clinical and MRI parameters between the groups, confirming equitable data distribution. To ascertain the OR and p-values for features that differentiate benign from malignant vertebral compression fractures (VCFs), both univariate and multivariate analytical approaches were systematically applied. The univariate analysis highlighted significant differences (P < 0.05) in gender, age, location, band pattern edema, anterior wedge deformity, and paraverteral mass between the two groups. Multivariate analysis identified band pattern edema (OR=0.125), anterior wedge deformity (OR=0.189), and paraverteral mass (OR=13.538) as independent risk factors for differentiating between benign and malignant VCFs (**Supplementary Table 1**).

**Table 1:** Baseline clinical and MRI features of patients in training and testing sets.

Clinical and MRI features	All (n=234)	Training cohort (n=164)	Testing cohort (n=70)	P
Age (year)	63.32 ± 13.44	60.97 ± 11.37	64.33 ± 14.14	0.008
Gender				0.979
Male	105(44.87)	32(45.71)	73(44.51)	
Female	129(55.13)	38(54.29)	91(55.49)	
Location				0.105
Cervical	8(3.42)	5(7.14)	3(1.83)	
Thoracic	83(35.47)	22(31.43)	61(37.20)	
Lumbar	143(61.11)	43(61.43)	100(60.98)	
Anterior wedge deformity				0.526
Absent	106(45.30)	29(41.43)	77(46.95)	
Present	128(54.70)	41(58.57)	87(53.05)	
Band pattern edema				0.438
Absent	167(71.37)	47(67.14)	120(73.17)	
Present	67(28.63)	23(32.86)	44(26.83)	
Paraverteral mass				0.954
Absent	222(94.87)	67(95.71)	155(94.51)	
Present	12(5.13)	3(4.29)	9(5.49)	
Diffuse signal change				0.591
Absent	76(32.48)	25(35.71)	51(31.10)	
Present	158(67.52)	45(64.29)	113(68.90)	
Pedicle/posterior element involvement				0.754
Absent	119(50.85)	34(48.57)	85(51.83)	
Present	115(49.15)	36(51.43)	79(48.17)	

**Table 2:** Performance comparison of different clinical models.

Model	AUC	95% CI	Accuracy	Sensitivity	Specificity	PPV	NPV
LR-training	0.820	0.761 ~ 0.880	0.671	0.131	0.990	0.889	0.658
SVM-training	0.775	0.702 ~ 0.848	0.762	0.770	0.757	0.653	0.848
KNN-training	0.818	0.760 ~ 0.877	0.659	0.082	1.000	1.000	0.648
RandomForest-training	0.808	0.746 ~ 0.870	0.762	0.770	0.757	0.653	0.848
ExtraTrees-training	0.822	0.763 ~ 0.880	0.671	0.131	0.990	0.889	0.658
XGBoost-training	0.808	0.746 ~ 0.870	0.762	0.770	0.757	0.653	0.948
LightGBM-training	0.797	0.735 ~ 0.860	0.628	0.000	1.000	0.000	0.628
MLP-training	0.789	0.725 ~ 0.834	0.762	0.770	0.757	0.653	0.848
LR-testing	0.794	0.696 ~ 0.892	0.700	0.125	1.000	1.000	0.687
SVM-testing	0.681	0.539 ~ 0.822	0.714	0.667	0.739	0.571	0.810
KNN-training	0.789	0.690 ~ 0.888	0.686	0.083	1.000	1.000	0.676
RandomForest-testing	0.778	0.676 ~ 0.880	0.714	0.667	0.739	0.571	0.810
ExtraTrees-testing	0.795	0.697 ~ 0.892	0.700	0.125	1.000	1.000	0.687
XGBoost-testing	0.778	0.676 ~ 0.880	0.714	0.667	0.739	0.571	0.810
LightGBM-testing	0.767	0.664 ~ 0.891	0.714	0.667	0.739	0.571	0.810
MLP-testing	0.749	0.638 ~ 0.860	0.714	0.667	0.739	0.571	0.810

The identified variables served as discriminators for differentiating between benign and malignant VCFs and were integral in the development of the clinical prediction model. The newly constructed framework exhibited strong predictive capability, yielding area under the curve (AUC) scores of 0.822 (95% CI: 0.763-0.880) and 0.795 (95% CI: 0.697-0.893) in respective training and testing datasets. Detailed findings from both cohorts are systematically cataloged in **Table 2** and **Supplementary Figure 1**, demonstrating consistent classification accuracy across different sample populations.

### 3.2 Feature extraction and model development

#### 3.2.1 Rad models

In the domain of Rad, an extensive set of 1835 manually derived features was initially extracted, which included 360 fundamental characteristics, 14 morphological descriptors, and a diverse array of textural attributes. Through a rigorous feature selection process, this collection was winnowed down to 15 highly interrelated features that were deemed most pertinent for subsequent analysis and the crafting of conventional Rad predictive models. The prognostic efficacy of different classifier amalgamations are delineated in **Table 3**. Among these, the ExtraTrees algorithm emerged as the most accurate predictor in the testing dataset, yielding an AUC of 0.801(95% CI: 0.693- 0.909). The ROC curve for this particular model is depicted in **Supplementary Figure 2**.

**Table 3:** Performance comparison of different Rad models.

Model	AUC	95%CI	Accuracy	Sensitivity	Specificity	PPV	NPV
LR-training	0.876	0.820 ~ 0.932	0.835	0.721	0.903	0.815	0.845
SVM-training	0.938	0.887 ~ 0.990	0.921	0.902	0.932	0.887	0.941
KNN-training	0.891	0.845 ~ 0.937	0.805	0.557	0.951	0.872	0.784
RandomForest-training	0.887	0.836 ~ 0.937	0.805	0.836	0.786	0.699	0.890
ExtraTrees-training	0.901	0.857 ~ 0.946	0.787	0.902	0.718	0.655	0.925
XGBoost-training	0.890	0.840 ~ 0.940	0.817	0.803	0.825	0.731	0.876
LightGBM-training	0.981	0.967 ~ 0.996	0.927	0.885	0.951	0.915	0.933
MLP-training	0.879	0.825 ~ 0.933	0.787	0.836	0.757	0.671	0.886
LR-testing	0.745	0.617 ~ 0.872	0.686	0.625	0.717	0.536	0.786
SVM-testing	0.773	0.652 ~ 0.894	0.757	0.500	0.891	0.706	0.774
KNN-training	0.751	0.641 ~ 0.861	0.686	0.557	0.951	0.872	0.784
RandomForest-testing	0.794	0.681 ~ 0.908	0.714	0.667	0.739	0.571	0.810
ExtraTrees-testing	0.801	0.693 ~ 0.909	0.743	0.792	0.717	0.594	0.868
XGBoost-testing	0.685	0.553 ~ 0.818	0.657	0.375	0.804	0.500	0.712
LightGBM-testing	0.768	0.657 ~ 0.880	0.700	0.708	0.696	0.548	0.821
MLP-testing	0.689	0.547 ~ 0.832	0.714	0.500	0.826	0.600	0.760

#### 3.2.2 2.5D DL models

**Figure 3:**
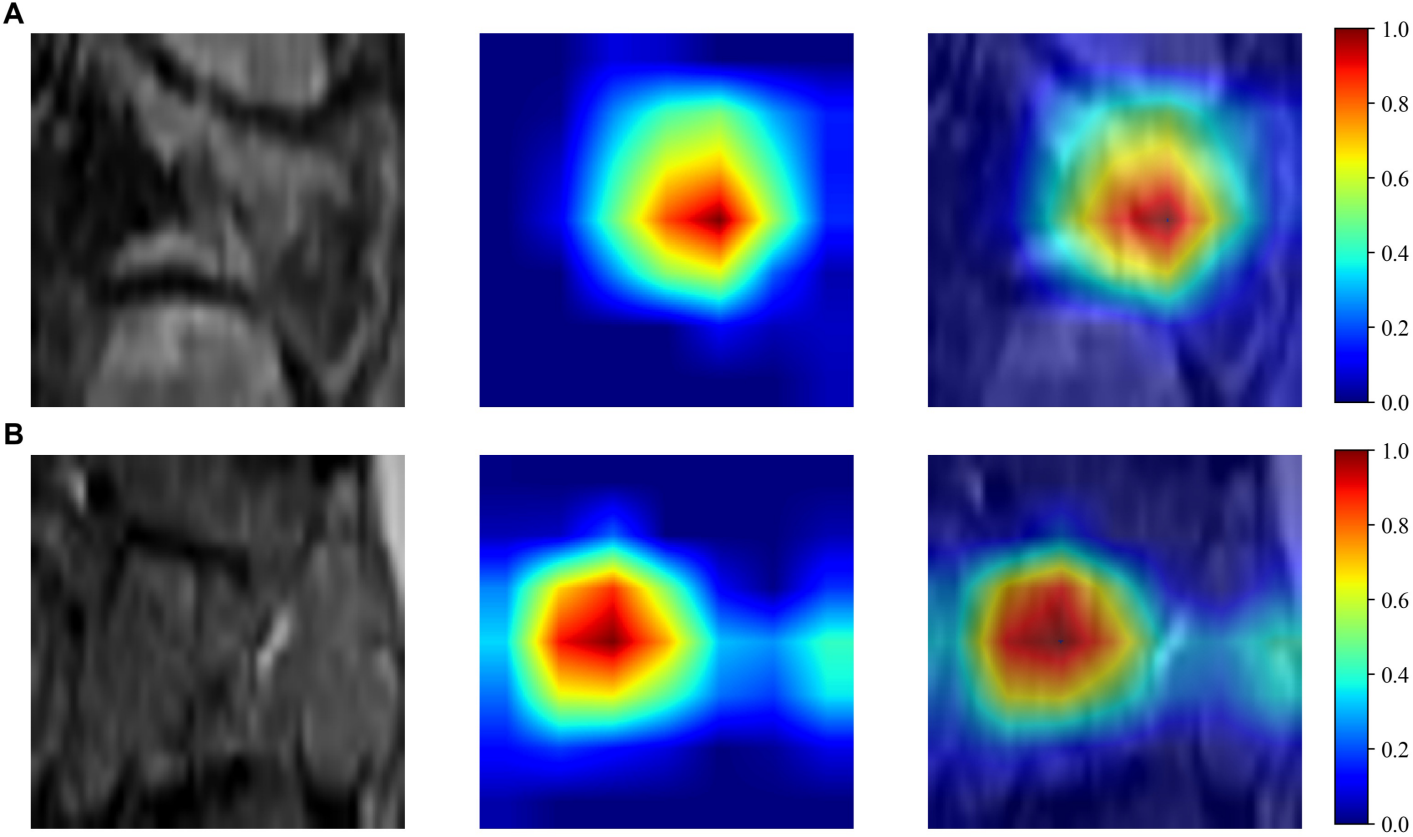
Grad-CAM visualizations for benign **(A)** and malignant **(B)** VCFs.

The Resnet50 architecture demonstrated a higher level of performance relative to the ExtraTrees algorithm when employing clinical and traditional Rad features, The proposed model exhibited excellent discriminative capability, as evidenced by an AUC value of 0.805 (95% CI: 0.690-0.921) in the independent testing cohort. To enhance our understanding of the ResNet50 model’s decision mechanism, we employed Gradient-weighted Class Activation Mapping (Grad-CAM), an advanced visualization technique that provides intuitive insights into the neural network’s feature attention patterns during classification. Grad-CAM provides a qualitative localization map, highlighting the critical regions that contribute to the classification outcome. During the analytical process, the output generated by the final convolutional layer within the last residual block was visualized with semi-transparency, as depicted in **Figure 3**.

#### 3.2.3 Feature fusion models

**Figure 4:**
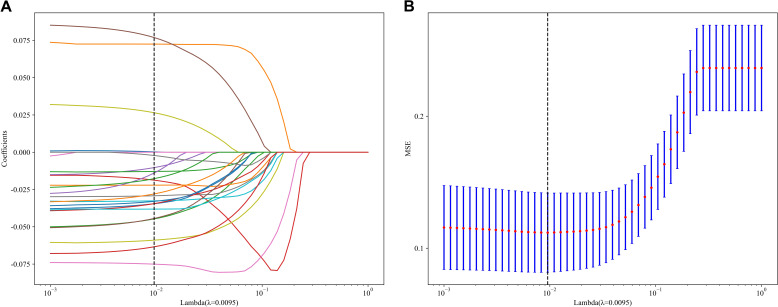
Fusion feature selection using LASSO **(A)** and the histogram of the feature importance score **(B)** based on the selected features. The optimal λ value of 0.0095 was selected.

**Figure 5:**
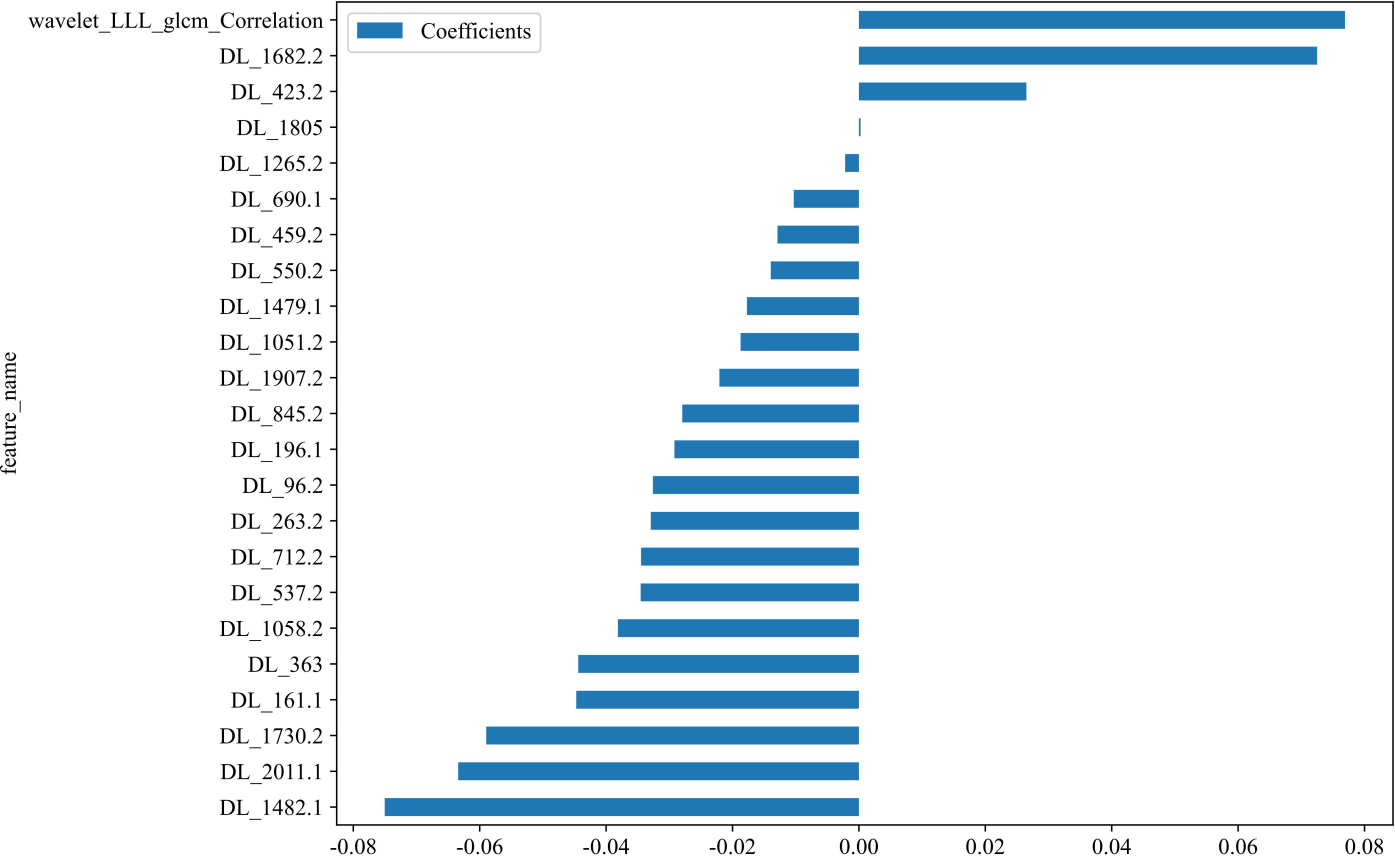
The selected fusion features and corresponding coefficients.

In the context of the training dataset, a selection of 23 Rad and DL features, characterized by nonzero coefficients, were identified to construct the DLR score through the application of a LASSO logistic regression model. The coefficients, along with the mean standard error derived from 5-fold cross-validation and the ultimate values of the selected nonzero coefficients, are presented in **Figures 4** and **5**. The resultant DLR score is presented below:

DLR_score = 0.3719512195121951 -0.034529 * DL_537.2 + 0.072502 * DL_1682.2 -0.044722 * DL_161.1 -0.018719 * DL_1051.2 -0.010304 * DL_690.1 + 0.076911 * wavelet_LLL_glcm_Correlation -0.075029 * DL_1482.1 -0.029188 * DL_196.1 -0.058964 * DL_1730.2 -0.032595 * DL_96.2 + 0.000263 * DL_1805 -0.022073 * DL_1907.2 -0.012883 * DL_459.2 -0.034471 * DL_712.2 -0.013942 * DL_550.2 -0.044407 * DL_363 -0.002176 * DL_1265.2 + 0.026500 * DL_423.2 -0.038139 * DL_1058.2 -0.032927 * DL_263.2 -0.027950 * DL_845.2 -0.017713 * DL_1479.1 -0.063406 * DL_2011.1.

Following the integration of diverse classifiers to create DLR feature fusion models, their respective performances were assessed and summarized in **Table 4**. In the testing dataset, the ExtraTrees classifier exhibited superior performance, achieving an AUC of 0.828 (95% CI: 0.727–0.929), outperforming the ResNet50 model. The DLR model’s superior AUC (0.828 *vs*. 0.805 for ResNet50 alone) suggests that handcrafted radiomic features complement DL-derived features, which may not be fully captured by end-to-end DL. The corresponding ROC curve is illustrated in **Supplementary Figure 3**.

**Table 4:** Performance comparison of different DLR models.

Model	AUC	95% CI	Accuracy	Sensitivity	Specificity	PPV	NPV
LR-training	0.982	0.967 ~ 0.996	0.915	0.951	0.893	0.841	0.968
SVM-training	0.997	0.992 ~ 1.000	0.963	0.984	0.951	0.923	0.99
KNN-training	0.973	0.954 ~ 0.992	0.890	0.738	0.981	0.957	0.863
RandomForest-training	0.964	0.938 ~ 0.991	0.909	0.885	0.922	0.871	0.931
ExtraTrees-training	0.971	0.948 ~ 0.995	0.921	0.902	0.932	0.887	0.941
XGBoost-training	0.963	0.936 ~ 0.990	0.915	0.885	0.932	0.885	0.932
LightGBM-training	1.000	0.999 ~ 1.000	0.988	0.984	0.99	0.984	0.990
MLP-training	0.974	0.949 ~ 0.992	0.933	0.836	0.99	0.981	0.911
LR-testing	0.729	0.606 ~ 0.853	0.643	0.833	0.543	0.488	0.862
SVM-testing	0.745	0.608 ~ 0.881	0.757	0.708	0.783	0.630	0.837
KNN-training	0.785	0.660 ~ 0.910	0.786	0.500	0.935	0.800	0.782
RandomForest-testing	0.789	0.669 ~ 0.909	0.786	0.625	0.87	0.714	0.816
ExtraTrees-testing	0.828	0.727 ~ 0.929	0.743	0.708	0.761	0.607	0.833
XGBoost-testing	0.739	0.620 ~ 0.858	0.657	0.708	0.630	0.500	0.806
LightGBM-testing	0.731	0.596 ~ 0.866	0.771	0.542	0.891	0.722	0.788
MLP-testing	0.813	0.705 ~ 0.920	0.743	0.708	0.761	0.607	0.833

### 3.3 Nomogram development and model performance comparison

**Figure 6:**
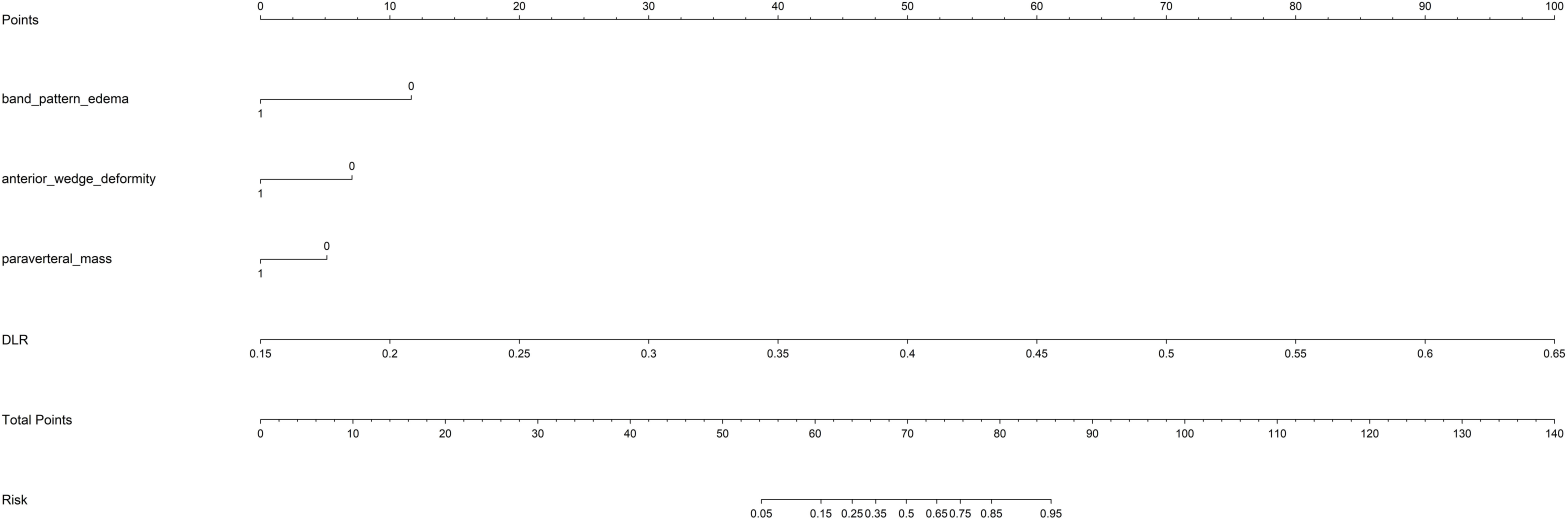
The DLRN for predicting VCFs. For clinical features in nomogram, 0 means ‘absent’, and 1 means ‘ present’ successively.

The DLR model exhibited superior efficacy in comparison to other models, prompting us to integrate pertinent clinical characteristics with its predictions to develop the ultimate combined model, which was adeptly depicted through a nomogram (DLRN). The nomogram revealed that the DLR factor was pivotal in distinguishing between benign and malignant VCFs (as shown in **Figure 6**).

**Figure 7:**
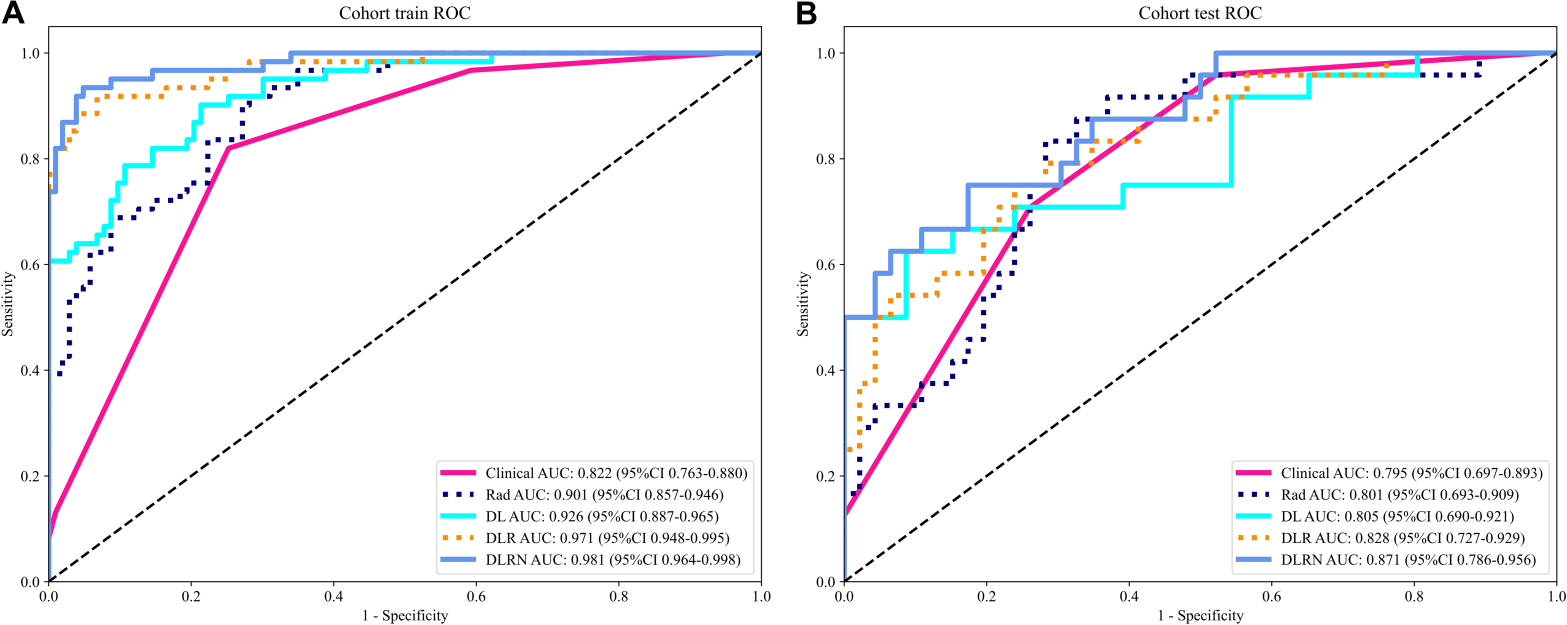
The ROC curves for different models in training cohort **(A)** and testing cohort **(B)**.

An overview of the performance metrics for a range of models, encompassing clinical, Rad, DL, DLR and DLRN features is provided in **Table 5**. Notably, as visualized in **Figure 7**, the DLRN model exhibited the most significant performance, achieving an AUC of 0.981 (95% CI: 0.964 - 0.998) in the training dataset and 0.871 (95% CI: 0.786 - 0.957) in the testing dataset.

**Table 5:** Performance comparison of different models.

Model	AUC	95% CI	Accuracy	Sensitivity	Specificity	PPV	NPV
Clinical-training	0.822	0.763 ~ 0.880	0.671	0.131	0.99	0.889	0.658
Rad-training	0.901	0.857 ~ 0.946	0.787	0.902	0.718	0.655	0.925
DL -training	0.926	0.887 ~ 0.945	0.823	0.885	0.786	0.711	0.920
DLR-training	0.971	0.945 ~ 0.995	0.921	0.902	0.932	0.887	0.941
DLRN -training	0.981	0.964 ~ 0.998	0.939	0.918	0.951	0.918	0.951
Clinical-testing	0.795	0.697 ~ 0.893	0.700	0.125	1.000	1.000	0.687
Rad-testing	0.801	0.693 ~ 0.909	0.743	0.792	0.717	0.594	0.868
DL -testing	0.805	0.690 ~ 0.921	0.800	0.583	0.913	0.778	0.808
DLR-testing	0.828	0.727 ~ 0.930	0.743	0.708	0.761	0.607	0.833
DLRN -testing	0.871	0.786 ~ 0.957	0.786	0.708	0.826	0.68	0.844

**Figure 8:**
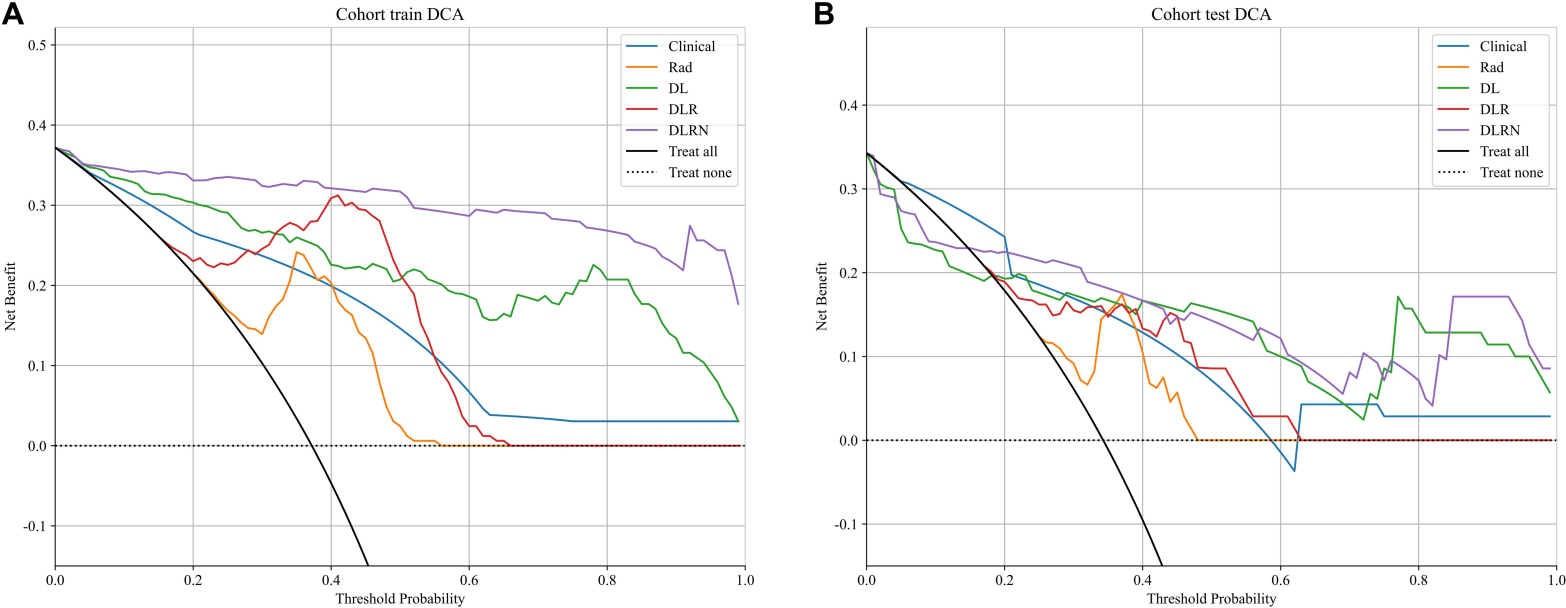
Different models’ DCA curves in training set **(A)** and testing set **(B)**.

To assess and compare the efficacy of the clinical, Rad, DL, DLR, and DLRN signatures, the Delong test was utilized, as illustrated in **Supplementary Figure 4**. The calibration curves displayed in **Supplementary Figure 5** indicate strong predictive accuracy of the DLRN model, with Hosmer-Lemeshow test results of 0.261 (training set) and 0.208 (testing set). Additionally, the DCA curves in **Figure 8** indicate that the DLRN model offers superior clinical utility compared to the Rad, DL, and DLR models.

## 4 Discussion

The results in current study indicated that 2.5D DL models surpassed traditional Rad models in distinguishing benign from malignant VCFs using MRI(AUC=0.805 *vs* 0.801). Furthermore, integration of the feature fusion DLR architecture yielded additional performance gains, achieving a superior AUC metric of 0.828. Both clinical features and MRI characteristics were found to contribute significantly to model development. Significantly, the DLRN model, which combined all relevant data, showed better performance (AUC=0.871). The DCA further underscored the potential advantages of utilizing the DLRN model, suggesting improved advantages for patients.

While prior research has described imaging characteristics of VCFs (16, 19), our systematic approach through univariate and multivariate logistic regression identified three clinically practicable predictors: anterior wedge deformity, absence of paravertebral mass, and bandlike edema. These findings corroborate established biomarkers while addressing the critical gap in quantitative statistical validation. Furthermore, the incorporation of various classifiers with clinical models yielded promising diagnostic results in our research. The clinical models exhibited robust predictive accuracy across both training and testing datasets, achieving AUC scores between 0.763 and 0.880 for the training dataset, and 0.697 to 0.892 for the testing dataset.

Rad is an evolving and fast-growing area that emphasizes the comprehensive extraction of quantitative features, revealing detailed textures invisible to human sight (19, 20). Various research endeavors have been undertaken to distinguish benign from malignant vertebral VCFs by extracting and examining imaging characteristics. To differentiate between benign and malignant VCFs, Zhang et al. (21) obtained Rad features from MRI scans of 479 patients and utilized seven different machine learning classifiers to distinguish between ambiguous VCFs. These models showed favorable efficacy in differentiating benign from malignant indistinguishable VCFs, and Gaussian naïve Bayes (GNB) model attained higher AUC (0.860). The incorporation of various classifiers with traditional Rad models also yielded promising diagnostic results in our research. Specifically, the training set exhibited an AUC within a certain range, while the testing set demonstrated an AUC within another specified range. While the training dataset achieved consistently high AUC scores between 0.857 and 0.946, the testing set showed a more moderate performance range from 0.693 to 0.909.

When compared to conventional machine learning methods, DL, with its multiple layers of artificial neurons, excels at extracting abstract and complex features from raw data, rendering its models exceptionally proficient in detecting sophisticated patterns and relationships within image data (22, 23). Spinal surgeons have shown considerable interest in the potential of DL models as a viable option for diagnosing malignant VCFs, offering an alternative to biopsy and postoperative pathology (24). Nonetheless, the classification capabilities of 2D DL models are constrained due to their neglect of tumor characteristics across adjacent slices (25). 3D segmentation leverages comprehensive spatial data of the tumor. However, it is crucial to acknowledge that such models require substantial computational power and large sample sizes to ensure adequate training (26). Our research employed a 2.5D technique that integrates data from three neighboring slices. This methodology capitalizes on the strengths of 3D data without sacrificing the correlational integrity between 2D slices, thereby addressing the shortcomings of both 2D and 3D frameworks. Utilizing the ResNet50 architecture, our study attained an AUC score of 0.805 within the testing set (95% CI: 0.690 - 0.921), demonstrating a significantly improved performance. The Grad-CAM visualization revealed that the model primarily concentrated on the tumor’s perimeters when making decisions. This alignment with clinical indicators enhanced the model’s interpretability.

Research has shown that models integrating DL characteristics with Rad features exhibit superior performance compared to models utilizing only one of these features in diverse clinical scenarios. Duan et al. (27) investigated various models, including DL, Rad, and their fusion (DLR), to distinguish malignant VCFs from those caused by osteoporosis. Notably, the DLR model demonstrated superior performance. Consistent with Duan et al’s findings, our findings indicated that fusion model (DLR AUC=0.828) surpassed both individual models.

Furthermore, choosing an effective and suitable classification model is essential for constructing reliable models. In the testing set of our study, which included clinical, Rad, and DLR models, the ExtraTrees classifier delivered outstanding results. By integrating extra randomness from the RandomForest approach, the ExtraTrees method successfully minimizes model variance and boosts its ability to generalize, rendering it exceptionally suitable for managing datasets (28).

The nomogram-based integration of multimodal features has enabled the development of clinically robust predictive frameworks (29, 30). The evaluation revealed enhanced diagnostic accuracy, as evidenced by the testing cohort’s AUC of 0.871 (95% confidence interval: 0.786–0.957). Notably, while individual models demonstrated satisfactory performance, our composite model synergistically combined DL with clinical parameters and Rad signatures, capitalizing on their complementary strengths to create a more clinically viable predictive tool. Importantly, this integrated framework suggests potential optimization of current clinical workflows by strategically reallocating computational resources without compromising diagnostic accuracy.

Compared with the most similar previously published article, our work has some similarities and differences with them. Prior studies have explored machine learning and imaging features for VCF differentiation. Thawait et al. (31) established MRI-based logistic regression models (training AUC: 0.872) using 34 handcrafted features, while Foreman et al. (32) developed a CT-focused 3D U-Net model (external AUC: 0.76). However, Flanders’ reliance on manual feature engineering limits generalizability, and Foreman’s CT-based approach sacrifices MRI’s superior soft-tissue contrast. To address these gaps, we propose a novel 2.5D deep learning framework integrating radiomics and MRI-specific features. By integrating 2.5D deep learning with radiomics, our study advances beyond Flanders’ feature engineering and Foreman’s CT-based models, offering a MRI-specific, interpretable nomogram for VCF differentiation. This approach balances discrimination power (testing AUC: 0.871), computational efficiency, and clinical utility.

Nonetheless, this study has several limitations. Initially, its retrospective nature could introduce bias in participant selection. The study’s participant selection was restricted to a single healthcare institution, resulting in limited external verification of findings. Subsequent research initiatives should prioritize multi-center collaborations to broaden demographic representation and strengthen the model’s clinical applicability. Moreover, our approach to extracting features and constructing a model was based only on traditional w-Dixon images with manually delineated ROIs, without incorporating other imaging sequences. Future work will concentrate on developing comprehensive multi-modal imaging models for detailed data collection and incorporating intelligent segmentation techniques based on deep learning to ensure higher delineation accuracy and consistency, consequently elevating diagnostic quality.

## 5 Conclusions

The present research indicated that the DLR model, which integrates feature fusion from MRI data, outperforms clinical models, traditional Rad models, and 2.5D DL models in differentiating benign from malignant VCFs. Furthermore, the incorporation of clinical and MRI parameters further boosts the efficacy of the DLRN. Ultimately, this approach has significant potential to support precision medicine initiatives in clinical practice. Future work will focus on multi-center validation and incorporation of multi-modal imaging to further strengthen the model’s clinical applicability.

## Data Availability

The original contributions presented in the study are included in the article/**Supplementary Material**. Further inquiries can be directed to the corresponding author.
