# Correction: MRI-based 2.5D deep learning radiomics nomogram for the differentiation of benign versus malignant vertebral compression fractures

**DOI:** 10.3389/fonc.2025.1632503

**Published:** 2025-06-11

**Authors:** Wenhua Liang, Hong Yu, Lisha Duan, Xiaona Li, Ming Wang, Bing Wang, Jianling Cui

**Affiliations:** Department of Radiology, Third Hospital of Hebei Medical University, Shijiazhuang, China

**Keywords:** radiomics, 2.5D deep learning, feature fusion, vertebral compression fractures, nomogram, magnetic resonance imaging

In the published article, there was an error in affiliation. Instead of “Department of Radiology, Third Hospital of Hebei Medical University, Shijiangzhuang, China”, it should be “Department of Radiology, Third Hospital of Hebei Medical University, Shijiazhuang, China”.

The original version of this article has been updated.

