# Supplementary material for: MRI-based 2.5D deep learning radiomics nomogram for the differentiation of benign versus malignant vertebral compression fractures
Source: Front Oncol. 2025 May 14;15:1603672. doi: 10.3389/fonc.2025.1603672 (PMC12116352; doi:10.3389/fonc.2025.1603672)
Supplement: Supplementary file 1 [file DataSheet1.docx]

Supplementary Material

**1. Clinical and MRI features**

**Supplementary Table 1.** Univariable and multivariable analysis of clinical and MRI features.

| Feature name | OR UNI | Lower 95%CI | Upper 95%CI | P | OR MULTI | Lower 95%CI | Upper 95%CI | P |
| --- | --- | --- | --- | --- | --- | --- | --- | --- |
| Band pattern edema | 0.048 | 0.014 | 0.157 | <0.05 | 0.125 | 0.034 | 0.460 | <0.05 |
| Anterior wedge deformity | 0.192 | 0.119 | 0.310 | <0.05 | 0.189 | 0.094 | 0.379 | <0.05 |
| Gender | 0.358 | 0.242 | 0.530 | <0.05 | 0.483 | 0.247 | 0.946 | 0.075 |
| Location | 0.823 | 0.744 | 0.910 | <0.05 | 1.506 | 0.957 | 2.370 | 0.137 |
| Diffuse signal change | 0.948 | 0.696 | 1.292 | 0.778 |  |  |  |  |
| Age | 0.992 | 0.988 | 0.996 | <0.05 | 0.996 | 0.977 | 1.014 | 0.707 |
| Pedicle/posterior element involvement | 1.257 | 0.866 | 1.824 | 0.312 |  |  |  |  |
| Paraverteral mass | 8.000 | 1.398 | 45.787 | <0.05 | 13.538 | 2.143 | 85.541 | <0.05 |

**2. Clinical models**


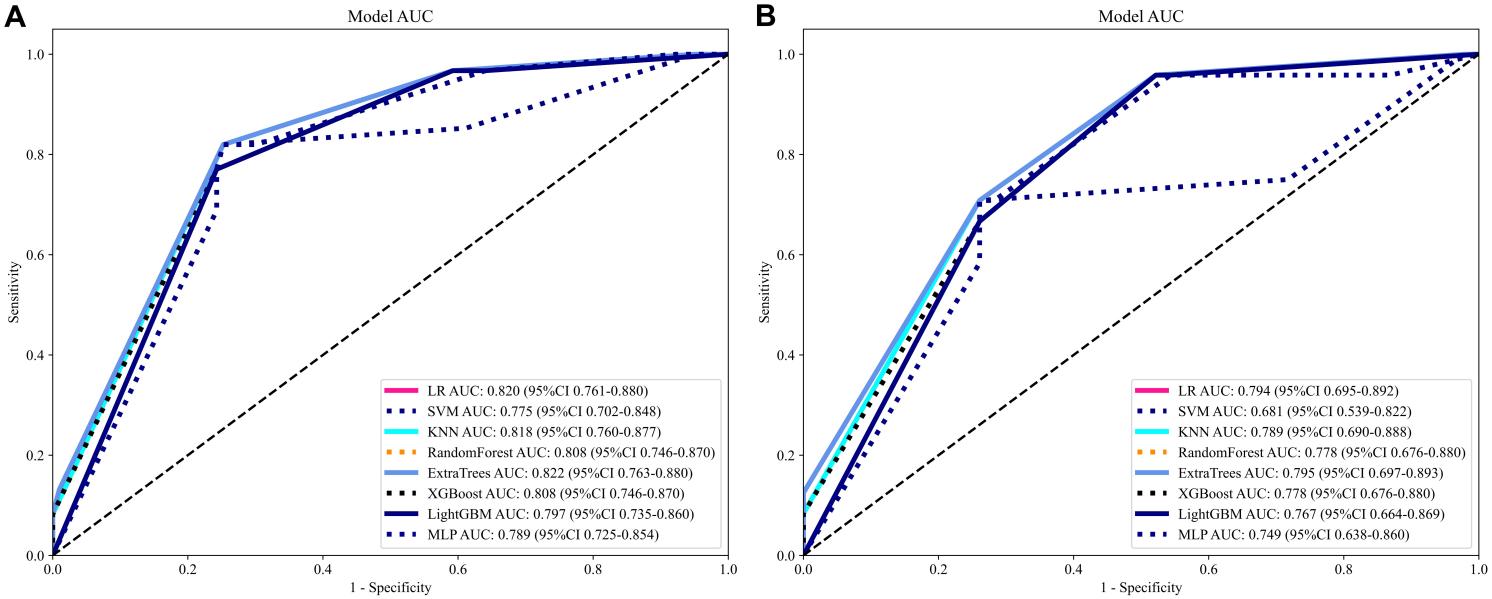


**Supplementary Figure 1.** ROC curves for clinical models in training set (**A**) and testing set (**B**).

**3. Rad models**


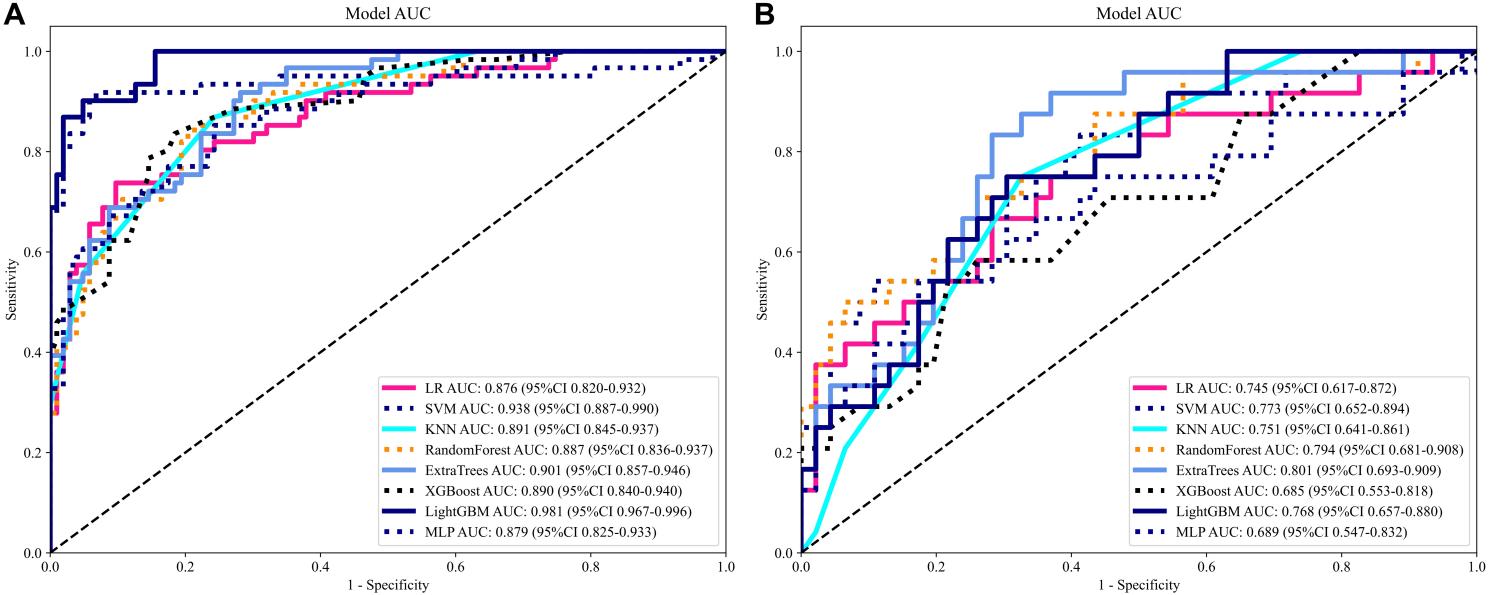


**Supplementary Figure 2.** ROC curves for Rad models in training set (**A**) and testing set (**B**).

**4.Deep learning radiomics (DLR) models**


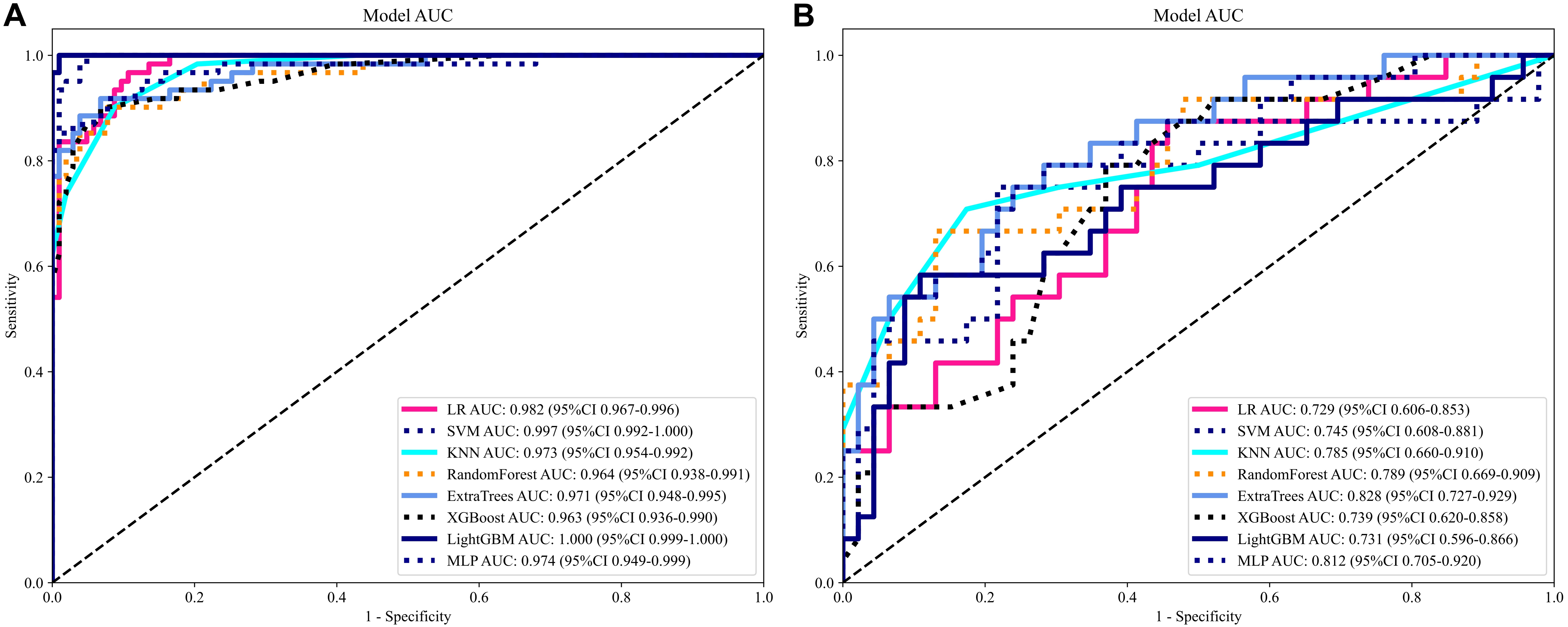


**Supplementary Figure 3.** ROC curves for DLR models in training set (**A**) and testing set (**B**).

**5.** **Delong Test**


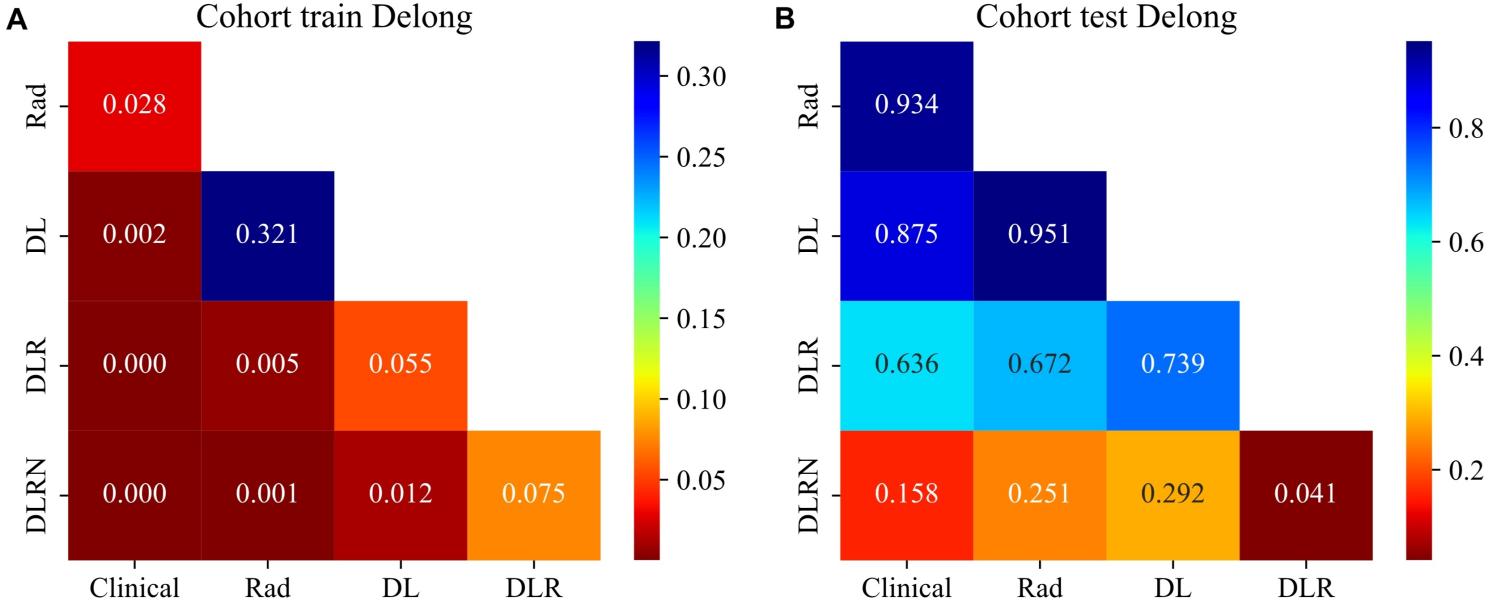


**Supplementary Figure 4.** Delong test in training set (**A**) and testing set (**B**).

**6. Calibration curves**

**
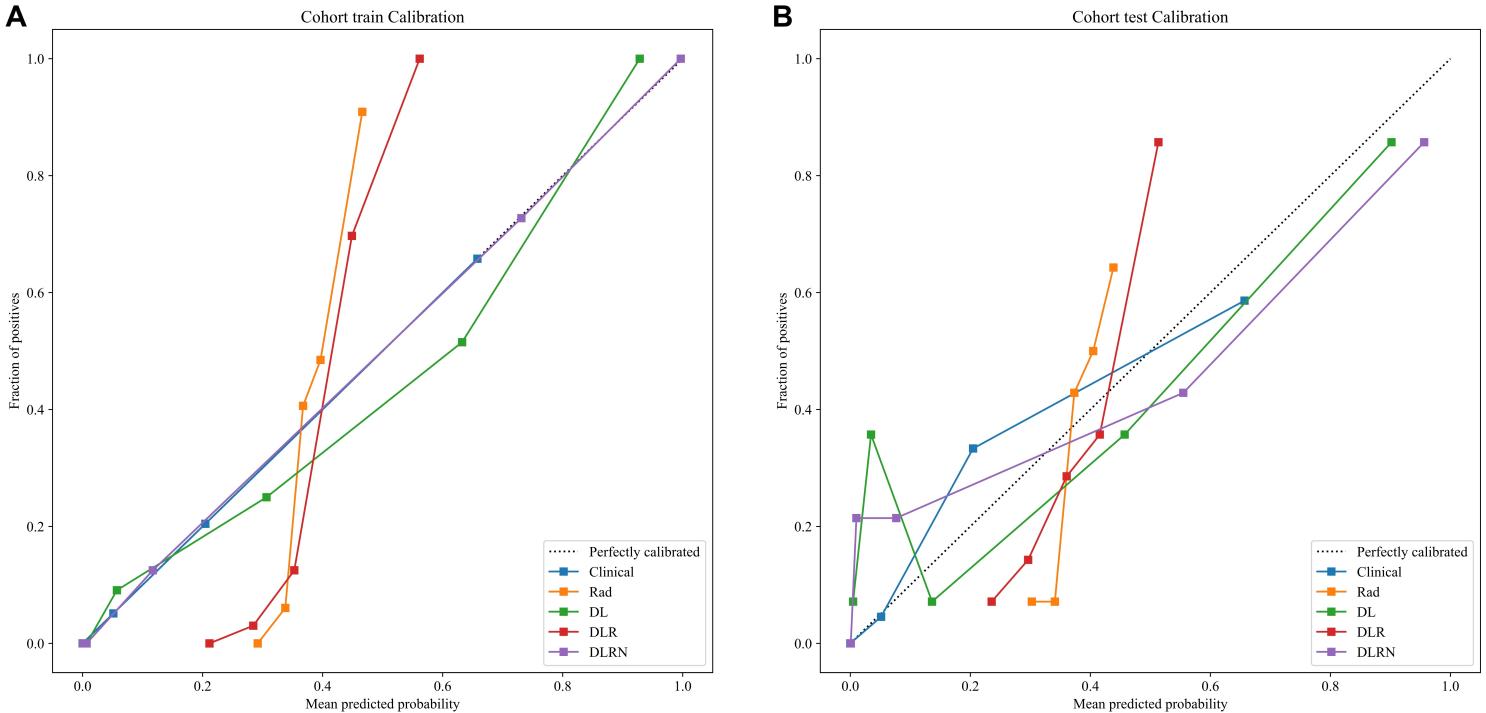
**

**Supplementary Figure** **5.** The calibration curves for different models in training set (**A**) and testing set (**B**).
